# Placing Local Aggregations in a Larger-Scale Context: Hierarchical Modeling of Black-Footed Albatross Dispersion

**DOI:** 10.1371/journal.pone.0153783

**Published:** 2016-04-28

**Authors:** P. E. Michael, J. Jahncke, K. D. Hyrenbach

**Affiliations:** 1 Hawai’i Pacific University at Oceanic Institute, Kalanianaole Hwy, Waimanalo, Hawai'i, 96795, United States of America; 2 Point Blue Conservation Science, 3820 Cypress Dr, #11, Petaluma, California, 94954, United States of America; University of Aveiro, PORTUGAL

## Abstract

At-sea surveys facilitate the study of the distribution and abundance of marine birds along standardized transects, in relation to changes in the local environmental conditions and large-scale oceanographic forcing. We analyzed the form and the intensity of black-footed albatross (*Phoebastria nigripes*: BFAL) spatial dispersion off central California, using five years (2004–2008) of vessel-based surveys of seven replicated survey lines. We related BFAL patchiness to local, regional and basin-wide oceanographic variability using two complementary approaches: a hypothesis-based model and an exploratory analysis. The former tested the strength and sign of hypothesized BFAL responses to environmental variability, within a hierarchical atmosphere—ocean context. The latter explored BFAL cross-correlations with atmospheric / oceanographic variables. While albatross dispersion was not significantly explained by the hierarchical model, the exploratory analysis revealed that aggregations were influenced by static (latitude, depth) and dynamic (wind speed, upwelling) environmental variables. Moreover, the largest BFAL patches occurred along the survey lines with the highest densities, and in association with shallow banks. In turn, the highest BFAL densities occurred during periods of negative Pacific Decadal Oscillation index values and low atmospheric pressure. The exploratory analyses suggest that BFAL dispersion is influenced by basin-wide, regional-scale and local environmental variability. Furthermore, the hypothesis-based model highlights that BFAL do not respond to oceanographic variability in a hierarchical fashion. Instead, their distributions shift more strongly in response to large-scale ocean—atmosphere forcing. Thus, interpreting local changes in BFAL abundance and dispersion requires considering diverse environmental forcing operating at multiple scales.

## Introduction

Terrestrial and marine ecologists have long quantified the spatial dispersion of organisms, defined by the changes in their occurrence and abundance across space, to gain insights into the ecological factors influencing species patchiness and structuring biological communities [1–4]. Ecological studies have assessed patchiness by comparing the observed spatial patterns to theoretical models of random and non-random dispersion [1, 4]. Researchers have also quantified the intensity (degree of aggregation) and the form (patch sizes) of spatial dispersion, to investigate the ecological processes influencing species distribution and abundance patterns [5–8].

Traditionally, seabird spatial dispersion is described in terms of occurrence, delineating species ranges, and abundance, identifying areas of aggregation [9–11]. Furthermore, the distribution of seabird counts across standardized sampling units (point counts or transects) is used to quantify the intensity and the form of their dispersion [6, 12, 13]. In particular, replicated surveys over seasons and years have facilitated the study of changing seabird dispersion during periods of contrasting oceanographic conditions and prey availability [7, 8, 14, 15]. Increasingly, these spatially-explicit perspectives of aggregation and predator-prey associations are being used to monitor changing marine ecosystems [16, 17].

Recent advances in oceanographic sampling and analytical methods have increased the understanding of the environmental drivers of seabird dispersion [18, 19], with direct conservation and management applications, [20, 21]. In particular, previous studies have quantified habitat associations [5, 13, 22], predator-prey relationships [7, 8, 23], and the scale-dependency [6, 12, 16] of seabird dispersion patterns. However, few analyses have evaluated the influence of environmental factors on seabird patchiness, as they relate to the degree of aggregation (intensity) and the form (patch sizes) of spatial dispersion.

We used replicate vessel-based surveys to explored the degree to which black-footed albatross (*Phoebastria nigripes*: BFAL) dispersion in the central California Current System (CCS) is influenced by local, regional and basin-wide patterns of ocean—atmosphere variability. More specifically, we focused on the patchiness of BFAL distributions to characterize those areas and habitat features where they form aggregations. This research builds upon previous findings that BFAL dispersion patterns differ across bathymetric domains: intense aggregation on the shelf (< 200 m depth), random scattering on the slope (200–2,000 m depth), and uniform abundance in oceanic waters (> 2,000 m depth) [24, 25]. To further investigate the drivers of BFAL patchiness, we identified those environmental variables most strongly associated with the intensity and the form of their spatial dispersion, using a hypothesis-driven model and an exploratory analysis.

## Methods

### Study region

**Fig 1:**
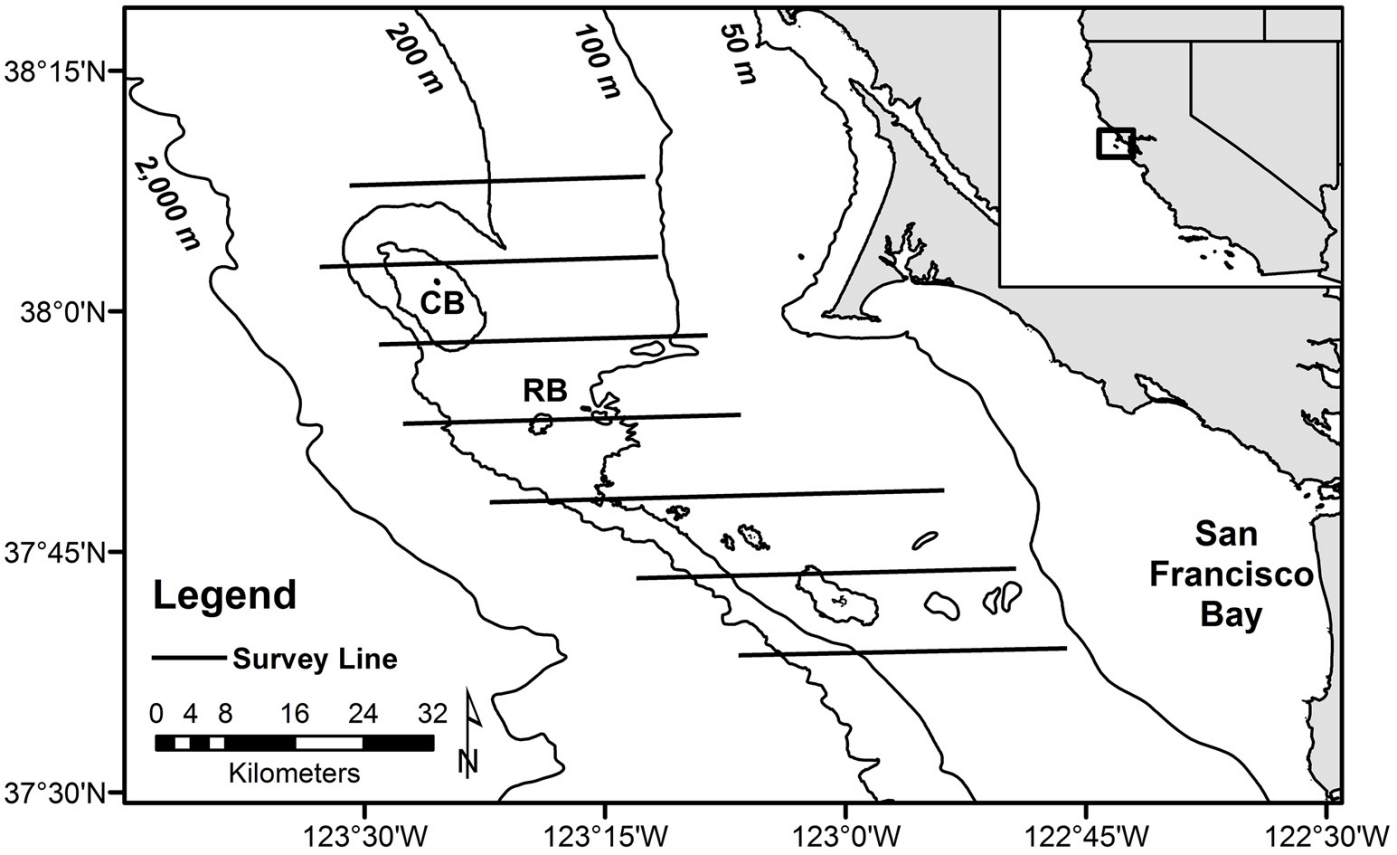
Map of study area showing the replicate transect lines surveyed and important bathymetric features. Cordell Bank (CB) and Rittenburg Bank (RB) are delineated by the extent of the 100 m isobath.

We studied BFAL dispersion within the productive continental shelf / slope system of central California over two breeding seasons (chick-rearing, post-breeding) using five years (2004–2008) of standardized vessel-based surveys collected by the ACCESS (Applied California Current Ecosystem Studies) partnership. This region includes complex physiographic (shelf break, banks, canyons) and hydrographic (upwelling plumes, fronts) features associated with highly productive habitats [26–28], which are exploited by locally-breeding and migratory upper-trophic marine predators [29, 30], including BFAL [24, 31]. A steep shelf-break / slope (200–2000 m depth) delineates the shallow continental shelf (< 200 m), which is broader to the south (~ 80 km) and narrower to the north (~ 45 km) (Fig 1).

This region is also characterized by strong seasonality in atmospheric and oceanographic conditions, which define three distinct oceanographic seasons: upwelling, oceanic, and Davidson [32, 33]. The upwelling season spans from early spring (mid-March) to late summer (mid-August), with the strongest upwelling occurring in May and June. Its onset is marked by an abrupt decrease in sea-surface temperature (SST) associated with the strengthening of upwelling-favorable winds from the northwest [34, 35].

### At-sea surveys and albatross dispersion

Trained ACCESS observers surveyed BFAL distributions using standardized strip transect methods [28, 36]. One to two observers counted BFAL during daylight hours from the flying bridge of three vessels (the 17.1m R/V John H. Martin, the 20.4 R/V Fulmar, and the 68.3m NOAA Ship McArthur II) with observer eye-heights of 5.3 m, 5.5 m, and 14.4 m, respectively, above the surface of the water as the vessel moved at a speed of 18–22 km h ^-1^, and recorded these observations in a field computer with a temporal resolution of 0.1 minute. Observers recorded every BFAL sighted within a 100 m to 300 m strip transect (depending upon the survey platform and viewing conditions) extending from abeam to 90° off of the side of the vessel with the best visibility (i.e., lowest glare).

Replicate cruises sampled seven parallel east—west survey lines, numbered sequentially from north to south, spanning from line 1 (north of Cordell Bank, 38° 8’ N) to line 7 (south of the Farallon Islands, 37° 34’ N), and extending from shallow coastal waters (50 m depth) within 15 km from shore, to the upper continental slope (1,084 m depth). All survey lines intersected the shelf break, with lines 2 and 3 crossing over Cordell Bank (CB, summit depth = 37 m) and line 4 crossing over Rittenburg Bank (RB, summit depth = 79 m, Fig 1). Yet, due to slight deviations in vessel course due to weather conditions, replicate surveys of the same line sampled bins of varying depth and location.

We restricted our analyses to survey lines that had been surveyed entirely and consisted of bins of standardized length and area, and discarded those lines interrupted by a gap in survey effort. Point Blue Conservation Science Geographic Information Systems (GIS) technicians binned the survey effort and BFAL sightings into 3 km intervals, with shorter bins resulting from survey interruptions at hydrographic stations and the end of the lines. We only considered bins between 1 and 3 km in length, and discarded those characterized by poor visibility conditions (strip width < 100 m), irrespective of their length.

### Environmental variables

We used 18 variables to characterize environmental conditions and temporal variability along each survey line (Table 1): (a) seasonal timing within a year (expressed using Julian day), and across years (expressed using cumulative Julian day since the start of the study), (b) static features (mean depth, latitude), (c) dynamic local hydrographic features (mean sea-surface temperature (SST), mean sea-surface salinity (SSS)), (d) dynamic regional atmosphere-ocean conditions (meridional (N-S) wind, zonal (E-W) wind, wind modulus, atmospheric pressure, change in atmospheric pressure, 6hr upwelling and monthly upwelling both north (39° N) and south (36° N) of the study area), (e) two basin-wide indices (Pacific Decadal Oscillation, PDO [37], http://jisao.washington.edu/pdo/PDO.latest and North Pacific Gyre Oscillation, NPGO [38], http://npgo.o3d.org/data/NPGO.txt). Finally, because the length of the survey transects can influence the form and intensity of the observed BFAL dispersion, we also considered the length of each line.

**Table 1:** Environmental variables evaluated in the models.

Variable	Data source	Interpretation
Temporal		-
Breeding Season (Julian day)	-	Intra-annual variation
Year (cumulative Julian day)	-	Inter-annual variation
Static		-
Mean Depth	Shipboard / CA DFG MR	Bathymetric domain
Mean Latitude	Shipboard	North—South gradient
Survey Line Length	Shipboard	Methodological biases
Dynamic		-
SST / SSS	Shipboard	Water mass
EW / NS Wind	PFEL	Wind direction
Wind Modulus	PFEL	Wind speed
Atmospheric Pressure	PFEL	Weather systems
Δ Atmospheric Pressure	PFEL	Movement of weather systems
6hr Upwelling Index (36°N, 39°N)	PFEL	Water Mass Advection
Monthly Upwelling Index (36°N, 39°N)	PFEL	Primary Productivity
Basin-Wide	-	-
PDO	http://jisao.washington.edu/pdo/PDO.latest	Broad-scale fluctuation in SST, SSH
NPGO	http://npgo.o3d.org/data/NPGO.txt	Broad-scale fluctuation in nutrients, salinity, SSH

CA DFG MR = California Dept. of Fish and Game Marine Region, SST (SSS) = sea-surface temperature (salinity), SSH = sea-surface height. Δ demotes change in a given variable. PFEL = Pacific Fisheries Environmental Laboratory. Upwelling indices: Monthly (36° N 122° W) and 6hr (39° N 125° W). PDO = Pacific Decadal Oscillation, NPGO = North Pacific Gyre Oscillation.

#### Static features

Point Blue Conservation Science GIS technicians derived the mean depth for each survey bin, from California Department of Fish and Game, Marine Region GIS lab 200m Exclusive Economic Zone (EEZ) bathymetric grids (ftp://ftp.dfg.ca.gov/R7_MR/BATHYMETRY/). The length of each survey line was calculated using the vessel’s navigational system, with an accuracy of 3–10 m. These data were used to calculate the position (latitude / longitude) of each survey bin centroid.

#### Local dynamic features

Point Blue Conservation Science GIS technicians calculated the mean SST and SSS for each survey bin, from the vessel’s underway data-logging system, with a spatial resolution from 3–10 m [28]. Whenever a survey bin did not contain any SST or SSS measurements, we linearly interpolated the missing values using the average of the two adjacent bins positioned immediately before and after along the track.

#### Regional dynamic features

We retrieved wind speed and upwelling indices from publicly available datasets compiled by the Pacific Fisheries Environmental Laboratory [39]. Wind-scape data involved meridional (N-S) and zonal (E-W) wind speed (m s^-1^, 0.25 degree latitude / longitude spatial resolution, 6hr temporal resolution), derived from blended satellite and buoy data. We calculated the absolute wind speed (modulus) from these two components using trigonometry. Atmospheric pressure data (millibars, 1 degree latitude / longitude spatial resolution, 6-hr temporal resolution) for the time of the survey and for the previous 24hr time period were retrieved from this database. Because moving weather systems influence seabird distributions (e.g., [40]), we calculated the change in atmospheric pressure (value during survey—value 24 hours before).

The upwelling data involved 6hr and monthly values (m^3^ s^-1^ 100 m coastline^-1^, 1 degree resolution) calculated at a location south (36° N 122° W) and north (39° N 125° W) of the study area. This approach provided the complete spatial coverage of the study area needed to identify the phasing and intensity of upwelling during cruises [29, 41]. Because many of these variables were expected to covary, we quantified the pair-wise cross-correlations of all the 6hr / daily environmental variables (Table 2). Otherwise, the correlations of the monthly upwelling / oceanographic indices have been previously reported [38, 42, 43].

**Table 2:** Cross-correlations of environmental variables measured along 41 survey lines.

	**SST**	**SSS**	**Depth**	**Depth Moran's I**	**Zonal Wind**	**Meridional Wind**	**Wind Modulus**	**6hr UW at 39N**	**6hr UW at 36N**	**Atm. Press**	**Δ Atm. Press.**	**Line Length**	**Lat.**	**BFAL Density**
**SST**	-	-0.415	0.043	-0.205	-0.572	0.275	-0.483	-0.240	-0.493	-0.245	0.335	-0.001	-0.114	-0.167
**SSS**	0.007	-	-0.099	0.206	0.136	-0.416	0.065	0.437	0.132	-0.248	-0.461	0.134	-0.088	0.222
**Depth**	ns	ns	-	0.261	0.169	-0.136	0.058	0.123	0.086	-0.097	-0.023	-0.799	0.708	0.101
**Depth Moran's I**	ns	ns	ns	-	0.202	0.021	-0.002	0.030	0.200	0.019	-0.057	0.162	-0.127	-0.099
**Zonal Wind**	< 0.001	ns	ns	ns	-	-0.495	0.550	0.333	0.760	0.489	-0.229	-0.154	0.125	-0.041
**Meridional Wind**	0.082	0.007	ns	ns	0.001	-	-0.447	-0.810	-0.520	-0.057	0.574	0.129	-0.095	-0.013
**Wind Modulus**	0.001	ns	ns	ns	< 0.001	0.003	-	0.415	0.416	0.152	-0.244	-0.138	0.127	-0.071
**6hr UW at 39 N**	ns	0.004	ns	ns	0.034	< 0.001	0.007	-	0.440	-0.253	-0.616	-0.134	0.135	-0.063
**6hr UW at 36 N**	0.001	ns	ns	ns	< 0.001	< 0.001	0.007	0.004	-	0.556	-0.187	-0.121	0.103	-0.245
**Atm. Press**	ns	ns	ns	ns	0.001	ns	ns	ns	< 0.001	-	0.310	0.048	-0.071	-0.185
**Δ Atm. Press.**	0.032	0.002	ns	ns	ns	< 0.001	ns	< 0.001	ns	0.048	-	0.037	-0.101	-0.001
**Line Length**	ns	ns	< 0.001	ns	ns	ns	ns	ns	ns	ns	ns	-	-0.681	-0.027
**Latitude**	ns	ns	< 0.001	ns	ns	ns	ns	ns	ns	ns	ns	< 0.001	-	0.082
**BFAL Density**	ns	ns	ns		ns	ns	ns	ns	ns	ns	ns	ns	ns	-

Pearson correlation coefficient (top diagonal) and the resulting p-value (bottom diagonal). ns = not significant (p > 0.10).

SST = sea-surface temperature, SSS = sea-surface salinity, UW = upwelling, Atm. Press. = atmospheric pressure, Δ Atm. Press. = change in atmospheric pressure, Lat. = latitude, BFAL = black-footed albatross.

#### Basin-wide indices

We used publicly-available monthly Pacific Decadal Oscillation (PDO) and monthly North Pacific Gyre Oscillation (NPGO) index values to characterize basin-wide oceanographic variability (Table 1). The PDO (NPGO) is the first (second) empirical orthogonal function, EOF1 (EOF2), of the analysis of the detrended sea-surface temperature anomalies (sea-surface height anomalies) north of 20 (25) degrees latitude north in the Pacific Ocean. The PDO is the dominant mode of variability north of 38° N, with positive values relating to anomalously warm SST in the CCS. The NPGO dominates south of 38° N, with positive values relating to anomalously high salinity, nutrients, and chlorophyll-a concentration [37, 38].

### BFAL dispersion

We calculated BFAL density (birds km ^-2^) in each survey bin and used two methods to quantify their spatial dispersion along those survey lines where BFAL were present. First, we computed the Green’s index (hereafter Gx):

$$Gx=\frac{\left(\frac{S^{2}}{\bar{x}}\right)-1}{\sum_{}^{}x-1}$$ (1)

where S^2^ is the variance of the densities for the line, $\bar{X}$ is the mean bird density for the line, and Σx represents the sum of the bird densities across the entire line. Values range from 1 (maximum aggregation: all birds in a single survey bin) to a small negative number equal to -1 * (Σx-1) ^-1^ (uniform distribution: same density of birds in each bin and variance of 0), with a value of 0 indicating a random distribution (variance equal to the mean; [44, 45]). To facilitate the interpretation of these analyses, we excluded those survey lines with a single BFAL sighting, since they would artificially yield a value indicative of maximum aggregation (Gx = 1).

We also assessed the form of BFAL dispersion (i.e., patch size) along every survey line using the Moran’s I index to quantify the spatial autocorrelation of the non-normal density data [46]. Moran’s I values range from -1 (negative autocorrelation, small patch size) to +1 (positive autocorrelation, large patch size), with values of 0 indicating a lack of autocorrelation (i.e., sample independence). To assess the potential influence of the spatial structure of the bathymetry on BFAL patchiness, we also quantified the Moran’s I index for the mean depth data (‘depth Moran’s I’) within the same survey bins used to quantify BFAL dispersion. We evaluated the Moran’s I using the Microsoft Office Excel RookCase add-in, and quantified its significance using Monte Carlo tests with 1,000 permutations[47]. Finally, to explore the influence of BFAL overall abundance on the intensity and the form of their spatial dispersion, we calculated BFAL density (number km ^-2^) along each survey line, by dividing the total number of birds sighted by the total area surveyed.

#### Data standardization

We calculated the mean value of each environmental variable along each survey bin, and standardized (mean = 0, S.D. = 1) the influence of variables with different ranges (i.e., atmospheric pressure, water depth) using Z scores, by subtracting the mean and dividing by the standard deviation of their respective distributions. This transformation ensured the normality of the data, thus facilitating the use of Pearson correlations and linear regressions [48].

### Environmental correlates

We investigated the influence of oceanographic and atmospheric variables on BFAL dispersion following two complementary approaches: a “hypothesis-driven” model and an “exploratory” analysis. The hypothesis-driven model incorporated *a priori* predictions from previous observations and sought to develop a hierarchical conceptual representation of the system dynamics [49]. More specifically, each connection within the hierarchy represented an explicit testable hypothesis, constrained by the inherent structure of the underlying conceptual model [50]. Conversely, the exploratory approach was not constrained by an *a priori* structure and afforded greater flexibility for investigating BFAL associations with a suite of cross-correlated variables [42]. Yet, the exploratory results must be interpreted with caution because they are based on correlational relationships [51].

**Fig 2:**
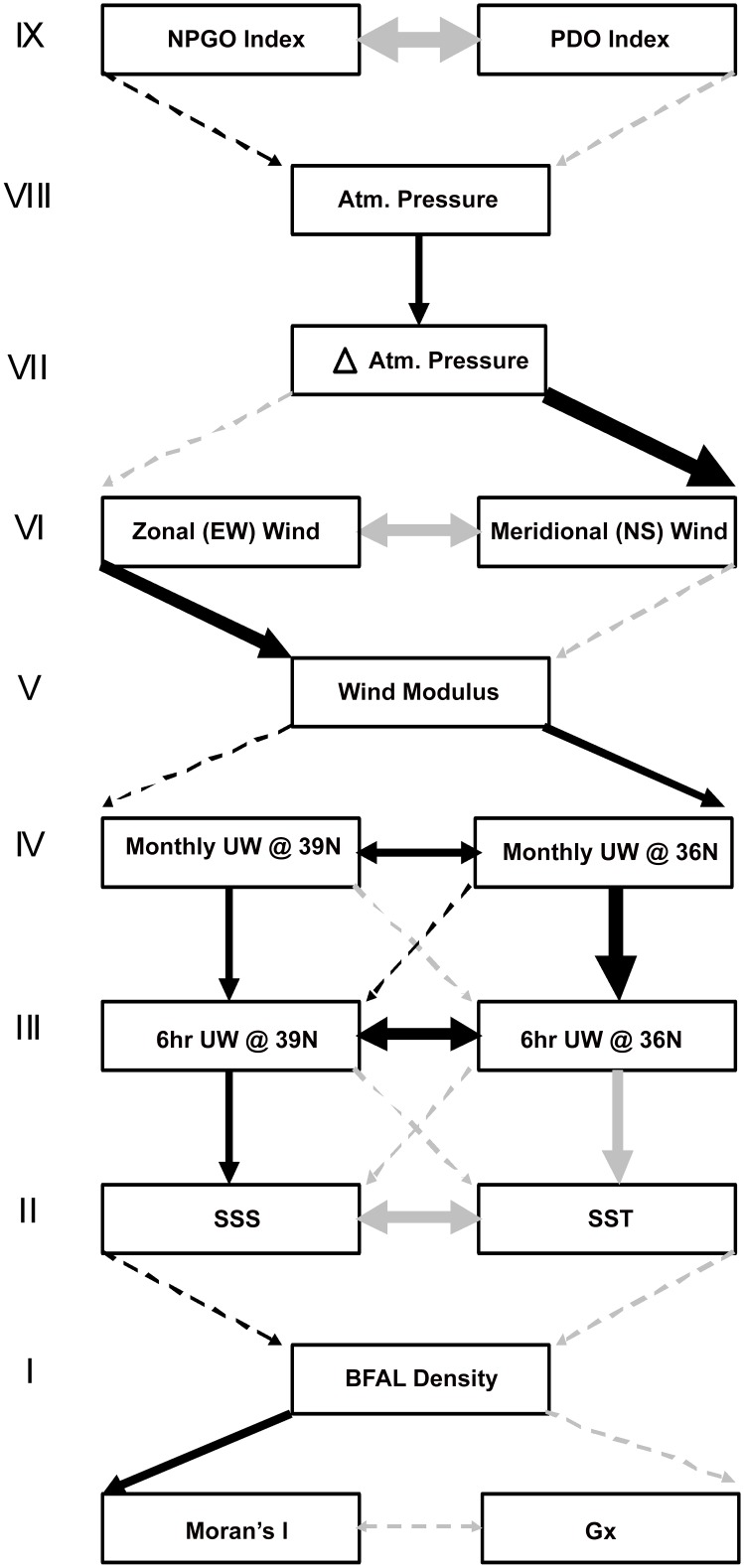
Results of the hypothesis-driven model, constructed using a hierarchy (nine levels, represented by roman numerals) of hypothesized drivers of BFAL dispersion. Pairwise correlations (double headed arrows within same level) denote covariation, and partial regressions (one-headed arrows across levels) indicate cause-effect relationships. Gx = Green’s Index of Dispersion, SST (SSS) = sea-surface temperature (salinity), UW = upwelling, and (Δ) Atm. = (change in) atmospheric. PDO = Pacific Decadal Oscillation, NPGO = North Pacific Gyre Oscillation. Arrow thickness indicates strength of the positive (black) or negative (grey) standardized path coefficients (in order of increasing weight, p ≥ 0.1, 0.05 to 0.01, 0.01 to 0.001, p ≤ 0.001). No p values between 0.1 and 0.05 were encountered. Dashed lines indicate non-significant (p ≥ 0.1) coefficients.

We used a simplified version of path analysis to evaluate the strength of the mechanistic connections in the hypothesis-driven model, by defining collinear relationships in a series of hierarchically organized variables [50, 52]. The path (Fig 2) began with both Gx and Moran’s I being subject to the influence of BFAL density (level I). The next level (II) of the model represented the local hydrographic variables (SST and SSS), assumed to be influenced by all other regional and basin-wide variables. The following level (III) included short-term (6 hr) regional upwelling at 36° N and 39° N, which influenced the SST and SSS and are, in turn, influenced by the levels above [35]. The next level (IV) included longer-term (monthly) regional upwelling at 36° N and 39° N, which represented the temporal integration of the more variable 6-hr observations [43]. The next four levels involved the wind modulus (V) which we derived directly from meridional and zonal wind speeds (VI), the change in atmospheric pressure (VII), and atmospheric pressure (VIII). The final level of this model (IX) included monthly values of the NPGO and PDO indices. All variables in the lower levels of the hypothesis model (I–VIII) were considered endogenous, theoretically having a mechanistic ‘driver’ in a higher level of the model. Conversely, the basin-wide indices were considered exogenous variables, capable of capturing other large-scale processes potentially affecting BFAL dispersion across the entire North Pacific. Because we hypothesized that the model captured the behavior of the system, regardless of the influence of temporal (season, year) variability, spatial (latitude, depth) gradients, and methodological biases (survey line length), we excluded these variables from this analysis.

Due to the large number of variables evaluated in the hypothesis-driven model (16), we only quantified the slope coefficients for variables within the same level (direct correlations) and with those from the level immediately above (direct causal relationship). Whenever multiple variables occurred in an upper level (i.e., level II above level I), we isolated the relationship of each lower-level variable (i.e., level I) with individual upper-level variables (i.e., level II) using partial regressions.

The exploratory analysis sought to identify the variables with the strongest explanatory power for both dispersion metrics. To this end, we performed a forward step-wise general linear model (GLM), and retained only those explanatory variables with significant relationships (p ≤ 0.1). We used the exploratory analysis to test three underlying assumptions of the hypothesis-driven model, by considering temporal variability (season, year), spatial gradients (latitude, depth), and sampling biases (survey line length). The selection of any of these variables in the exploratory GLM would highlight the hypothesis-driven model’s failure to account for important explanatory variables.

#### Statistical analyses

We performed the step-wise GLMs and Analysis of Variance (ANOVA) with Systat 11 [53], and the correlations and partial regressions using R[54], and assessed significance with alpha = 0.05. To address the potential false rejection of the null hypothesis (Type I error) due to multiple testing, we subjected the exploratory model results to the Bonferroni correction.

## Results

### At-sea surveys and albatross dispersion

We surveyed a total of 125 survey lines, during 19 cruises spanning the BFAL rearing period (April-June) and the post-breeding period (July, September-October) of five years (2004–2008). Of these 125 transects, 41 yielded no BFAL sightings and were thus excluded from subsequent analyses. An additional 17 transects with only one BFAL were also discarded because the sightings were perfectly aggregated (maximum patchiness, Gx = 1). Another 26 transects were discarded due to adverse weather conditions (strip width <100 m) or because they contained at least one short survey bin (< 1 km). The remaining 41 survey lines (Table 3) varied in length, with a mean = 35.07 km (S.D. = 5.09, range = 28.69 to 44.81), and yielded a total of 259 BFALs.

**Table 3:** Survey effort by month and season across all study years (2004–2008).

		# of		Total # of	
Season	Month	Years	Lines	Surveys	Lines
rearing	April	2	4	8	27
rearing	May	3	12	.	.
rearing	June	3	11	.	.
post-breeding	July	4	10	7	14
post-breeding	September	1	1	.	.
post-breeding	October	2	3	.	.
			**Total**	15	41

**Fig 3:**
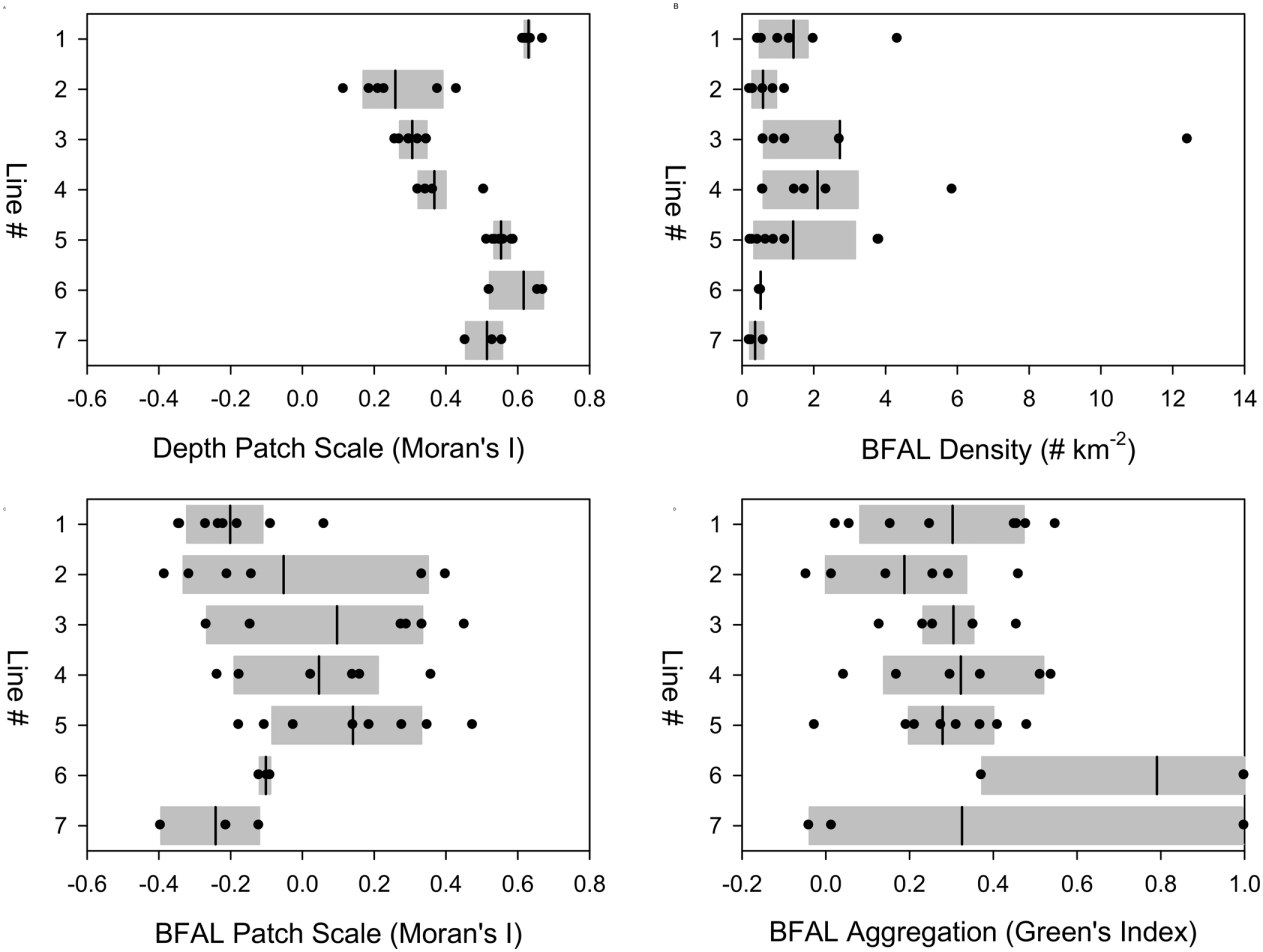
Boxplots of (a) Depth Moran’s I, (b) BFAL density, (c) BFAL Moran’s I, and (d) BFAL Green’s index by survey line, numbered from north (1) to south (7) (Fig 1). The vertical black lines denote the mean, the grey shading indicate the 25% and 75% of the distributions, and the circles show the individual observations.

First, we described the range of variability in BFAL density and dispersion (Gx and Moran’s I) across the study site. BFAL density along a survey line varied widely, with a mean = 1.48 BFAL km ^-2^ (S.D = 2.20, range = 0.22 to 12.42). BFAL dispersion along a survey line also varied widely, with mean Gx = + 0.32 (S.D. = 0.26, range = −0.05 to + 1.00) and mean Moran’s I = −0.02 (S.D. = 0.26, range = −0.39 to + 0.48) (Fig 3).

Next, we compared the patchiness of the underlying bathymetry across the seven survey lines. The patch scales of the bathymetric data, as indicated by the Moran’s I values, varied significantly by line (ANOVA, df = 6, 34, F = 36.759, p < 0.001) and the test residuals were normally distributed (Kolmorogov-Smirnov test, n = 41, Max-Diff = 0.128, p = 0.514) (Fig 3). Altogether, the ANOVA model captured a large proportion of the observed variability (r ^2^ = 0.866). Post-hoc pair-wise tests between all pairs of lines, conducted using Tukey tests, revealed two groupings: four lines (1, 5, 6, 7) were characterized by larger-scale patch sizes (higher Moran’s I), and three lines (2, 3 and 4) were characterized by smaller-scale patch sizes (lower Moran’s I). All 12 pair-wise comparisons between the lines in these two groups were significant (p ≤ 0.029), suggesting that the three lines (2, 3, 4) that crossed the two shallow banks (Cordell and Rittenburg) were characterized by a narrower bathymetric gradients than the shelf-break along the northern-most (line 1) and the southern-most lines (5, 6, 7).

Next, we compared BFAL abundance across the seven survey lines, after log transforming the data (y’ = log (y)) to achieve normality. BFAL densities did not vary significantly by line (ANOVA, df = 6, 34, F = 1.989, p = 0.095) and the residuals were normally distributed (Kolmorogov-Smirnov test, n = 41, Max-Diff = 0.099, p = 0.813). Overall, the ANOVA only captured a small proportion of the observed variability (r ^2^ = 0.260). While BFAL densities were higher, on average, along the central lines of the study area (3, 4 5), they were highly variable, ranging over two orders of magnitude (0.21–12.42 BFAL km ^-2^) (Fig 3). In particular, the two southern-most lines (6, 7) were characterized by consistently low densities (< 1 BFAL km ^-2^).

The BFAL patchiness, as indicated by the Moran’s I, did not vary significantly by line (ANOVA, df = 6, 34, F = 2.260, p = 0.061) and the test residuals were normally distributed (Kolmorogov-Smirnov test, n = 41, Max-Diff = 0.079, p = 0.962) (Fig 3). While the BFAL patch sizes were larger along the lines with the highest densities (3, 4, 5), the ANOVA was not significant and only captured a small proportion of the observed variability (r ^2^ = 0.285). Nevertheless, this result highlights the influence of BFAL density on patchiness, and suggests that larger BFAL patches form in areas of aggregation. Interestingly, the two southern-most lines (6, 7) were characterized by negative Moran’s I values, indicative of very small patch sizes; likely arising from few isolated sightings.

Finally, the intensity of BFAL dispersion, as indicated by Green’s Index, was significantly different across the survey lines (ANOVA, df = 6, 34, F = 2.411, p = 0.048), and the test residuals were normally distributed (Kolmorogov-Smirnov test, n = 41, Max-Diff = 0.083, p = 0.939) (Fig 3D). Yet, only 5 of the 21 pair-wise comparisons using Tukey tests yielded significant results (p < 0.05). The Green’s Index values along line 6 were larger than along the other northern lines (1, 2, 3, 4, 5), the ANOVA captured a low proportion of the observed variability (r ^2^ = 0.298). Once more, the two southern-most lines (6, 7) stood apart, with occasionally high values (Gx = 1), indicative of perfect aggregation (all BFALs sighted within a single survey bin).

Overall, the BFAL Green’s Index and the BFAL Moran’s I calculated for the same survey lines were not significantly cross-correlated (Pearson correlation, n = 41, r = −0.188, p > 0.20), underscoring the complementary perspectives afforded by these two indices of dispersion. Furthermore, these two dispersion metrics were related to different variables: BFAL patch sizes (Moran’s I) were positively correlated with log-transformed BFAL density (Pearson correlation, n = 41, r = + 0.375, 0.02 > p > 0.01), suggesting that denser BFAL concentrations formed larger patches. The intensity of BFAL dispersion (large Green’s Index) was positively correlated with the patchiness of the depth data (Moran’s I) (Pearson correlation, n = 41, r = + 0.317, 0.05 < p < 0.02), suggesting that BFALs became more aggregated in areas of broader bathymetric gradients.

#### Environmental correlates

The hierarchical hypothesis-driven model captured several anticipated pair-wise associations between the environmental variables, both within a single level or across levels (Fig 2). There were four significant intra-level relationships: (a) the strong negative correlation between SST and SSS (level I), (b) the positive correlation of 6hr northern / southern upwelling (level III), (c) the positive correlation of northern / southern monthly upwelling (level IV), and (d) the negative correlation between NPGO and PDO (level IX).

The hypothesis-driven model also captured nine inter-level relationships, five of which involved upwelling: (a) lower SST was associated with increased 6hr upwelling at 36° N (levels II and III), (b) higher SSS was associated with increased 6hr upwelling at 39° N (levels II and III), (c) monthly upwelling and 6hr upwelling at 36° N were positively correlated (levels III and IV), (d) monthly upwelling and 6hr upwelling at 39° N were positively correlated (levels III and IV), and (e) wind modulus was positively correlated with monthly upwelling (levels V and IV) to the south (36° N) of the study area.

Additionally, five significant inter-level relationships involved wind speed and atmospheric pressure. During our study, meridional wind tended to be negative (northerly) and zonal wind tended to be westerly (positive). Thus, the two wind components had different correlations with overall speed, quantified by the wind modulus. While the zonal wind and modulus were positively cross-correlated, the meridional wind and the modulus were negatively cross-correlated (levels VI and V). Moreover, increases in atmospheric pressure were associated with weaker northerly wind (levels VII and VI). Finally, atmospheric pressure was positively correlated with increasing atmospheric pressure (levels VIII and VII).

The exploratory analysis also documented significant cross-correlations between the environmental variables, both within and across hierarchical levels (Table 2), which justified the use of step-wise regressions. Overall, nine environmental variables were significantly associated with BFAL dispersion: four for BFAL Gx, three for BFAL Moran’s I, and two for BFAL density (Table 4). All of the test residuals were normally distributed (Kolmogorov-Smirnov one sample test) for BFAL Gx (max. dif. = 0.093, p = 0.873, n = 41), BFAL Moran’s I (max. dif. = 0.14, p = 0.45), depth Moran’s I (max. dif. = 0.09, p = 0.90, n = 41), or BFAL density (max. dif. = 0.19, p = 0.09, n = 41).

**Table 4:** Exploratory results of forward stepwise general linear models (GLMs) of metrics of black-footed albatross (BFAL) dispersion.

Dispersion Metric	Adjusted r^2^	Step #	Variable	Standardized Coefficient	p Value
Gx	0.379	1	Zonal (EW) Wind	+0.451	**0.001**
		2	Monthly UW @ 39N	-0.306	0.02
		3	Latitude	-0.550	0.004
		4	Depth	+0.378	0.04
Moran's I	0.172	1	BFAL Density	+0.321	0.03
		2	Line Length	+0.359	0.02
		3	Depth Moran's I	-0.280	0.05
BFAL density	0.154	1	PDO index	-0.410	0.009
		2	Atm. Pressure	-0.260	0.09

The adjusted (multiple) r^2^ accounts for the number of parameters included in the model and indicates the strength of the association of the sum of the variables included with each dispersion metric. Step # = the sequential selection of the strongest variable in forward stepwise GLMs, where ‘1’ indicates the variable with the most explanatory power, ‘2’ the second most after the first (‘1’) was removed, and so on. UW = upwelling, PDO = Pacific Decadal Oscillation. Standardized coefficients indicate the strength and direction of the relationship of each variable with a given dispersion metric. Significant p values after applying the Bonferroni correction are bolded.

More intense BFAL aggregations (larger Gx values), were associated with positive zonal (westerly) wind, weaker monthly upwelling at 39° N, deeper water, and lower latitude. These results suggest that BFALs were more strongly aggregated along the shelf-break, especially along the southern portion of study area. Larger BFAL patch sizes (larger Moran’s I) were positively associated with BFAL densities, smaller bathymetric patch sizes (smaller depth Moran’s I), and longer survey lines. In turn, BFAL densities were significantly higher during periods of low PDO index values.

## Discussion

Our analyses indicate that BFAL dispersion off central California varies spatially and temporally, in relation to a broad range of local, regional and basin-wide environmental variables. First, the intensity of BFAL dispersion (Gx index) was related to latitudinal and on—off shore gradients, the speed of westerly wind, and the monthly upwelling north of the study area (39° N). Second, the spatial scale of BFAL patches (Moran’s I) was influenced by their overall density within the study area, and by the underlying bathymetric structure (Table 4).

These results build upon the previous understanding of BFAL distributions off central California [24, 25] by addressing two novel aspects of their spatial dispersion (intensity and form), and by considering a suite of larger-scale (regional and basin-wide) environmental drivers, involving upwelling dynamics and atmospheric conditions. In particular, the significant influence of the PDO and upwelling north of the study area underscore the influence of remote atmospheric and oceanographic drivers on local BFAL distributions within West Coast sanctuaries.

Nevertheless, while we explored these relationships within the framework of a hierarchical path analysis, the resulting BFAL responses to environmental variability need to be interpreted with caution because they are mere cross-correlations subject to the, limited range of environmental variability we sampled during our four-year study.

### Data exploration and hypothesis testing

We used two complementary approaches to model the relationships of environmental variables with BFAL dispersion, which allowed us to test specific hypothesis-driven predictions and to explore empirical cross-correlations. Despite capturing some of the hypothesized mechanistic associations, the hypothesis-driven model failed to produce a ‘path’ linking BFAL dispersion to the anticipated causal factors (Fig 2). This failure is highlighted by the lack of significant standardized path coefficients between several consecutive levels of the hierarchical model: SST / SSS and BFAL density (levels II and I), and NPGO / PDO indices and atmospheric pressure (levels IX and VIII).

Yet, while the hypothesis-driven model did not link all the consecutive levels in the hypothesized hierarchy, it was consistent with the prevailing understanding of the physical forcing in the central California Current System: a negative relationship between SST and SSS characteristic of upwelling systems [35], a positive relationship between concurrent northern and southern upwelling [43], and a negative relationship between zonal and meridional winds [55]. The hypothesis-driven model also underscored the disparity in the intensity (Gx) and the form (Moran’s I) of BFAL dispersion, as evidenced by their opposing relationship with BFAL density (negative for Gx, positive for Moran’s I, Fig 2).

The inclusion of several additional variables to quantify temporal / spatial variability and potential methodological biases (survey line length), allowed us to assess their influence on BFAL dispersion. While BFAL density was not influenced by these variables, BFAL dispersion intensity (Gx) and form (Moran’s I) were significantly associated with two variables (latitude and depth) and one variable (survey line length), respectively (Table 4). Yet, despite identifying ‘significant’ relationships among the metrics of BFAL dispersion and these environmental variables, the exploratory analysis accounted for a small proportion of the overall observed variation in Gx (37.9%), Moran’s I (23.2%), and density (15.4%).

### Green’s index of dispersion

The intensity of BFAL dispersion was positively correlated with water depth (Table 4), which in the study site corresponds to the continental shelf-break and the upper slope (200–2000 m), especially during and periods of stronger westerly wind. These results reinforce the significance of the shelf-break as a site of BFAL aggregation off central California [24, 25], and suggest that these aggregations shift shoreward during periods of enhanced westward wind. During our surveys, we documented substantial variability in westerly winds (mean = 4.18 ± 2.22 S.D, range = 0.11 to 9.63 m s^-1^), indicative of the alternation of upwelling favorable and relaxation events. As noted previously, wind speed plays an important role in albatross flight energetics [56, 57] and habitat associations [58, 59].

BFAL dispersion was also more intense along the southern lines (lower latitude) and during periods of weaker monthly upwelling north of the study area (39° N) (Table 4). Together, these results suggest that BFALs aggregate downstream from the upwelling centers, in areas outside of the influence of the cold plumes of recently upwelled water, as has been previously documented [60]. Off Oregon, BFAL abundance in late spring (May–June) and summer (July–August) of 2000 was positively correlated with wind speed and areas of high sea-level height and warm-water, with the highest densities occurring within 30 km offshore of the center of the coastal upwelling jet [60]. Similarly, BFAL occurrence off central California during the spring (May–June) of 1997 was significantly higher in warmer and clearer waters, offshore of the influence of the coastal upwelling plumes [24].

Accordingly, we hypothesize that during periods of increased upwelling in the northern portion of the study region, BFAL distributions spread out over a larger area, spanning the offshore boundary of the coastal upwelling plumes. Conversely, during periods of lower upwelling, BFAL distributions shift onshore and become focused along the convergence zones outlining the upwelling plumes [30, 60]. While strong westerly winds are regionally associated with increased upwelling [28], the directionally opposing aggregation patterns could relate to the mismatch in temporal scales between the wind data (6hr) and the upwelling data (monthly).

Moreover, the higher degree of BFAL aggregation to the south (lower latitude) is likely the result of the underlying north-south habitat gradients influencing BFAL distribution patterns in the study area (Table 2). Because the southern lines sampled a broad shallow (< 200 m) shelf (Fig 1), the higher BFAL densities in the deeper shelf-break and slope waters (200–2000 m) were sampled by few bins at the offshore edge of the survey lines, resulting in intense BFAL aggregations. Additionally, dense BFAL aggregations occasionally occur in shallow water, further contributing to highly aggregated distributions in the southern part of the study area [24, 25].

### Moran’s I

BFAL patch sizes were most strongly correlated to BFAL density. In particular, the survey lines with the higher BFAL density yielded the larger patch sizes because they contained a larger number of consecutively occupied survey bins. Moreover, the underlying bathymetric habitats influenced BFAL patch sizes. Due to the extensive continental shelf to the south of the study area, the southernmost survey lines tended to be longer and disproportionately sampled the continental shelf (< 200 m depth), whereas the northern lines sampled different bathymetric habitats (shelf—shelf-break—upper slope) more equally (Fig 1). Thus, BFAL distributions were more aggregated over the deeper water overlying the offshore edge of the southern lines and more spread out along the northern lines.

### BFAL density

The exploratory analysis revealed that BFAL abundance was negatively correlated with the PDO index (Table 4). In fact, the PDO index decreased and BFAL density increased concurrently during the study period (2004–2008). Thus, it is possible that conditions associated with a low PDO index, namely positive SST anomalies in the central and western north Pacific [37], could increase fledging / juvenile survival or shift BFAL distributions from the western to the eastern North Pacific. Another non-exclusive possibility could be that relatively neutral to cool conditions in the study area could increase prey availability, attracting greater BFAL densities. It remains unclear whether this increase was caused by a short-term re-distribution, a longer-term population increase, or both. Yet, because non-breeding BFAL can travel across the north Pacific Ocean, covering 1000s of km in a matter of days [61], our finding suggest that local BFAL dispersion off central California is influenced by broad-scale ocean-atmosphere variability. Nevertheless, this correlational result needs to be interpreted with caution [51]. Thus, additional surveys during a period of increasing PDO are needed to test whether BFAL densities would decline concurrently.

We also documented a marginally significant (p = 0.09, Table 4) association of higher BFAL density with lower atmospheric pressure. This result suggests that more BFAL flew through the survey lines during periods of lower atmospheric pressure, resulting in higher densities. In particular, if BFAL ride along the edge of low pressure cells, the movement of these systems through the study area may result in higher BFAL densities. In a previous satellite telemetry study of a single BFAL, which crossed two low pressure cells, its movement vectors were significantly cross-correlated with concurrent wind speed and direction [62], suggesting this bird was using the pressure cell to travel with the wind.

Thus, while BFAL movements are likely influenced by wind speed and direction, it is unclear whether their local density would be expected to increase ahead of a low pressure cell. There is an ongoing debate regarding the influence of atmospheric pressure on albatross movements, specifically if their flight patterns draw them away [57] or towards [63] high pressure cells, and if the low wind-speeds in these cells act as ‘traps’ [64] or afford enhanced foraging opportunities [63]. Thus, our results add information to this debate, which merits further investigation.

The hypothesis model also yielded unexpected, albeit non-significant, directional relationships of BFAL density with two hydrographic variables: SST (negative, p > 0.5, df = 39) and SSS (positive, p > 0.2, df = 39). This suggests that BFAL were associated with recently upwelled water. Though previous surveys off California have documented the opposite pattern, with BFAL associations with relatively warm SST [24] and downwelling [30, 60], these studies addressed different temporal and spatial scales.

## Conclusions

In summary, we documented key similarities and differences between two distinct metrics used to quantify BFAL dispersion, which largely reinforced earlier findings of the distribution patterns in this highly-mobile seabird [24, 25]. Specifically, the intensity and the form of BFAL dispersion were correlated with different environmental drivers: BFAL Green’s Index was associated with strong westerly wind and low PDO index, and BFAL Moran’s I was associated with large BFAL density and small bathymetric patches (depth Moran’s I). These findings suggest that BFAL dispersion relates more strongly to regional-scale (6hr wind) and broad-scale (PDO) variability than to local-scale conditions (SST, SSS). Thus, localized habitat-use studies should consider the potential influence of environmental variables encompassing a broad range of temporal and spatial scales, and how observations in the non-breeding season can be impacted by conditions in other portion of the species range and the previous breeding season. Furthermore, these results suggest that BFAL do not respond to oceanographic variability in a strictly hierarchical fashion, whereby the influence of large-scale drivers is mediated by local-scale processes. Rather, their distributions shift in response to basin-wide and regional forcing in the atmosphere and the ocean. Thus, interpreting local changes in their abundance and distribution requires considering distant forcing mechanisms.
